# Underlying disease determines the risk of an open abdomen treatment, final closure, however, is determined by the surgical abdominal history

**DOI:** 10.1007/s00068-019-01205-2

**Published:** 2019-08-26

**Authors:** Steffi Karhof, Mark Haverkort, Rogier Simmermacher, Falco Hietbrink, Luke Leenen, Karlijn van Wessem

**Affiliations:** Department of Surgery, University Medical Centre Utrecht, Utrecht, The Netherlands

**Keywords:** Open abdominal treatment, Temporary abdominal closure, Open abdomen, Delayed fascial closure

## Abstract

**Introduction:**

Temporary abdominal closure is frequently used in several situations such as abbreviated surgery in damage control situations or when closing is impossible due to organ distention or increased abdominal pressure. The ultimate goal is to eventually close the fascia; however, little is known about factors predicting abdominal closure. The purpose of this study was to identify characteristics associated with the need for open abdomen as well as indicating the possibility of delayed fascial closure after a period of open abdominal treatment.

**Methods:**

A retrospective review of all patients that underwent midline laparotomy between January 2008 and December 2012 was performed. Both factors predicting open abdominal treatment and possibility to close the fascia afterwards were identified and analyzed by univariate and multivariate analyses.

**Results:**

775 laparotomies in 525 patients (60% male) were included. 109 patients (21%) had an open abdomen with a mortality rate of 27%. Male gender and acidosis were associated with open abdominal treatment. In 54%, the open abdomen could be closed by delayed fascial closure. The number of laparotomies both before and during temporary abdominal treatment was associated with failure of closure.

**Conclusion:**

In this study, male sex and physiological derangement, reflected by acidosis, were independent predictors of open abdominal treatment. Furthermore, the success of delayed fascial closure depends on number of abdominal surgical procedures. Moreover, based on our experiences, we suggest to change modalities early on, to prevent multiple fruitless attempts to close the abdomen.

## Introduction

Temporary abdominal closure (TAC) as a regular step in surgical treatment has increasingly been accepted over the past decades. There are three main indications for leaving the abdomen open after an index operation; first of all, leaving an abdomen open can be necessary in critically ill patients as part of damage control surgery (DCS). This abbreviated surgery limits operating time in favor of physiological recovery in ICU. Another reason for leaving the abdomen open in seriously ill patients is the development of abdominal compartment syndrome (ACS), where distention of organs, from any origin, mostly due to resuscitation, can lead to increased intra-abdominal pressure, often preventing closure of the abdominal wall for mechanical reasons. Besides that, there are patients in whom it is not desirable to close an abdomen, because of local abdominal disease, for example, in patients in whom an early second look is warranted to re-evaluate the intra-abdominal contents.

Historically, patients with a septic abdomen who underwent a planned re-laparotomy because of alleged gross contamination used to be another indication of open abdominal treatment [1–6]. However, a randomized clinical trial comparing on-demand with planned re-laparotomy strategy in patients with severe peritonitis did not reveal any differences on death or major peritonitis-related morbidity between both groups [7]. Hence, there is no reason anymore for leaving the abdomen open after abdominal sepsis when the fascia can be immediately closed.

If temporary closure is warranted, there are several techniques to temporarily close the abdomen, although consensus is lacking [1, 4, 8–10]. Ideally, TAC techniques should, amongst other features, protect the bowel and avoid further fascial edge retraction, prevent ACS, limit contamination, prevent third space fluid losses, avoid formation of adhesions, provide support for ventilation and should be cost effective [2, 4]. Final goal of temporary abdominal closure after cessation of its pathophysiologic origin is delayed primary closure within a reasonable window rather than deliberately ending up in a ventral abdominal hernia [1]. Previous studies suggest closing the abdomen within 8 days, since after this period, a significant increase in complications has been found [1, 3]. Little is known about the factors that influence and predict successful delayed primary closure [11], although previously it was shown that duration of treatment as well as the physiological state of the patient influences outcome in this patient population [12]. Also, the indication of abdominal surgery and the reason for the open abdomen are associated with the odds to eventually close the abdomen, with previous studies suggesting abdominal sepsis has worse outcome on closure rates [5, 6].

The goal of this study was to identify patient and laparotomy characteristics associated with both the need for an open abdominal treatment and characteristics indicating the possibility of delayed fascial closure, after a period of open abdominal treatment.

## Materials and methods

A retrospective study of all patients who underwent a laparotomy in our department of surgery in a University teaching hospital between January 2008 and December 2012 was performed. University Medical Center Utrecht is a large teaching hospital and the only Level-1 trauma center in the province of Utrecht. It covers the central region of the Netherlands with a relatively small, but densely populated service area of 1500 km^2^ and approximately 1.3 million residents.

All planned and emergency laparotomies by midline incision in adult patients were included. All laparoscopic procedures (approximately 500 annually) performed during the same period were not considered in this analysis; however, converted laparoscopic procedures to a laparotomy were included. Exclusion criteria were all other abdominal incisions than midline. Data were collected from computerized medical records through ICD codes. Demographical data including gender, age, BMI, ASA classification, comorbidities including history of smoking or alcohol abuse, and indication for laparotomy were recorded, as well as interventional data (timing, closure technique, open abdominal treatment including its indication and duration of TAC). Furthermore, physiological data such as pH, lactate and base deficit were collected. Mortality and morbidity, e.g., development of entero-atmospheric fistula (EAF) or ventral hernias, were evaluated for all patients during follow-up at our hospital. CT scans were not part of standard postoperative care. Data on morbidities were collected either during hospital stay or during follow-up in clinic, with a standard follow-up of 6 weeks after surgery and further visits at physician’s discretion.

### Surgical technique of temporary abdominal closure

**Fig. 1:**
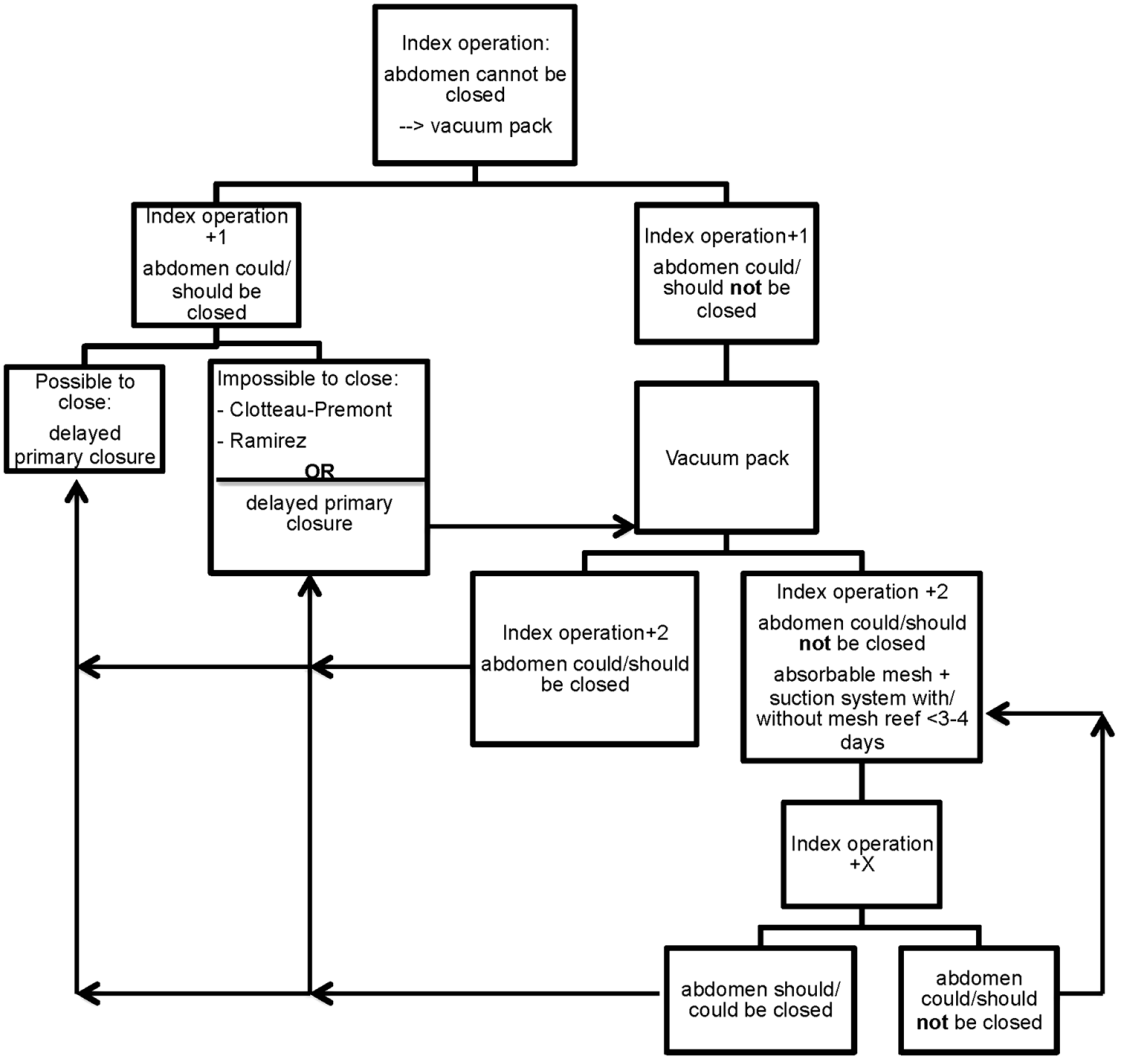
Algorithm to cover the open abdomen

In our hospital, a modified version of the vacuum pack to temporarily cover the open abdomen is used. This method was first described by Barker et al. [13] and uses multiple gauzes or a sterile surgical gown, wrapped in OpsiteTM (Smith & Nephew Inc, St. Petersburg, FL, USA) adhesive film to cover the abdominal contents. Over this pack, two holed drains are placed which is then covered with another Opsite adhesive film. The drains are connected with a Y piece and attached to a negative pressure suction pump creating a vacuum. Typically, after 24–72 h the VAC-pack will be removed for second-look surgery and possible fascial closure. When it is not possible or not favorable to close the abdomen, the same TAC technique will be used again. When delayed primary closure is not deemed feasible in the near future, a mesh to temporarily cover the abdomen can be used. This decision, including the timing to do so, is based on the clinical situation and at the surgeon’s discretion. In trauma patients a dedicated flowchart is used to aid in delayed fascial closure after damage control surgery (Fig. 1).

### Definitions of abdominal closure

Abdominal closure was divided into three categories. When the fascia was closed at the first intervention it was defined as ‘primary closure’, when the fascia was closed after a period of TAC, it was defined as ‘delayed fascial closure’ and when closure of the fascia was not possible and the wound healed over a mesh, it was called ‘secondary healing’ (or definitive open abdomen).

### Statistical analysis

All statistical analyses were conducted using IBM SPSS Statistics 22.0 (IBM Corporation, Armonk, NY, USA). All continuous non-parametric variables were reported in medians with interquartile ranges. Discrete variables were displayed as proportions. Bivariate analysis was performed using the *χ*² and Fisher’s exact test, respectively. Kruskal–Wallis one-way analysis of variance test was applied for comparisons between more than two independent groups. All variables that were tested in univariate analysis were also evaluated in a multivariate logistic regression analysis. These variables were analyzed with both forward and backward stepwise selection to identify independent risk factors for failure to close the abdomen. These outcomes were presented as odds ratios and 95% confidence interval. *p* values below 0.05 were considered statistically significant.

## Results

Between January 2008 and December 2012, a total of 525 patients underwent 775 laparotomies. The majority of patients were male (60%) with a median age of 61 years (Table 1). Three hundred seventy-one patients (71%) had at least one co-morbidity. Most patients had previous abdominal surgery (267; 52%) or cardiovascular history (197; 39%). The indication for laparotomy varied. The most common cause for surgery was gastro-intestinal in 593 patients (76.5%), followed by 122 patients (15.7%) after trauma and the remaining 60 patients (7.7%) had a laparotomy for vascular surgery (Table 2). There were 35 patients (7%) in whom a planned re-laparotomy was performed: 17 patients had a second look after abdominal packing, 11 patients for evaluation of on-going ischemia after bowel ischemia and 7 patients underwent second-look surgery for abdominal sepsis.

**Table 1:** Patient demographics

Patient demographics	*N* (%), total *n* = 525
Sex, male (%)	313 (60)
Age	61 (48–70)
BMI	24.4 (21.7–27.7)
ASA classification	2 (2–3)
Comorbidities	
Diabetes (total *n* = 514), yes (%)	72 (14)
Cardiovascular (total *n* = 511), yes (%)	197 (39)
Pulmonary (total *n* = 520), yes (%)	83 (16)
History of abdominal surgery (total *n* = 512), yes (%)	267 (52)
Total patients with comorbidities (%)^a^	371 (73)
Smoking (total *n* = 339), yes (%)	107 (32)
Alcohol abuse (total *n* = 341), yes (%)	115 (34)

All data are expressed in median (IQR) or absolute numbers (%)

^a^Unknown in 14 patients

**Table 2:** Laparotomy characteristics and risk for temporary open abdomen

	Total *N* = 775	Primary closure *N* = 666	TAC *N* = 109	*p* value	Odds ratio	95% CI
Indication for laparotomy						
Trauma	122	80 (12%)	42 (38%)	**< 0.001**	NS	
Gastro-intestinal^a^	593	542 (81%)	51 (47%)	**< 0.001**	0.47	0.24–0.88
Vascular	60	44 (7%)	16 (15%)	**0.006**	NS	
Timing of surgery	Acute 632	530 (79.6%)	102 (93.6%)	**< 0.001**	NS	
	Planned 143	136 (20.4%)	7 (6.4%)			
Gender: male	456	372 (55.9%)	84 (77.1%)	**< 0.001**	3.39	1.67–6.87
Previous abdominal surgery	392	355 (53.3%)	37 (33.9%)	**0.001**	NS	
ASA classification^b^	2 (1)	2 (1)	2 (1)	0.451	NS	
pH^b^	7.32 (0.18)	7.34 (0.16)	7.29 (0.19)	**< 0.001**	0.087	0.01–0.76
Lactate^b^	3.2 (3.95)	2.55 (2.98)	4.3 (4.2)	**0.003**	NS	
Base deficit^b^	− 6.35 (7.7)	− 5.2 (7.88)	− 7.25 (7.32)	**0.001**	NS	

All data are expressed in median (IQR) or absolute numbers (%)

All variables with a *p* value < 0.05 were considered significant and are in bold

*NS* not statistically significant, *TAC* temporary abdominal closure

^a^Gastro-intestinal surgeries include surgeries for abdominal sepsis and obstruction

^b^Values presented as median (IQR)

In most patients (416; 79%), the abdomen could be primarily closed, leaving a total of 109 patients (21%) for temporary abdominal closure (TAC). In 45 patients (41%), the abdomen was left open on account of DCS in trauma patients. In 37 patients (34%), the reason for TAC was abdominal sepsis, with the remaining 27 (25%) due to ACS (Table 2). The majority of patients (102; 93.6%) in whom the abdomen could not be primarily closed underwent surgery in an acute setting (Table 2).

When comparing characteristics of the patients in whom the abdomen was primarily closed to the TAC group in univariate analysis, significant indicative parameters were indications for laparotomy (*p* ≤ 0.001 for trauma and gastro-intestinal and *p* 0.006 for vascular surgery), male gender (*p* ≤ 0.001), previous abdominal surgery (*p* = 0.001), pH (*p* = 0.003), lactate (*p* ≤ 0.001) and base deficit (*p* = 0.001) (Table 2). When comparing all these variables (indication for laparotomy, timing of surgery, gender, previous abdominal surgery, ASA classification, pH, lactate and base deficit) in a multivariate analysis, only gastro-intestinal pathology as indication for laparotomy, gender and pH remained as independent factors associated with open abdominal treatment. Further, male patients had a 3.4-time higher chance of open abdominal treatment than female patients (Table 2). Of all patients following laparotomy the median hospital length of stay was 23 days, with a significant longer stay for patients following TAC of 46 days (Table 3). The same goes for ICU length of stay which was 11.5 days for patients following TAC and 0 days for patients following primary closure (Table 3). Complication rates were comparable in both groups with a mortality rate of 45% in patients following primary closure and 32% in patients who underwent open abdomen treatment (Table 3).

Twenty-nine patients died before the abdomen could be closed (27%): nine patients following DCS, ten after abdominal sepsis and ten treated for ACS. From the remaining surviving 80 patients, 43 could be managed by delayed fascial closure (54%). Four of them had their abdomen closed by mesh-mediated VAC closure and in one patient the abdomen was closed using the component-separation method as described by Ramirez [16].

The open abdomen of the remaining 37 patients (46%) was treated by secondary healing (Table 4).

**Table 3:** Outcome parameters

	Total *N* = 775	Primary closure *N* = 666	TAC *N* = 109	*p* value
Hospital length of stay^a^ (days)	23 (35)	20 (29)	46 (41)	**< 0.001**
ICU length of stay^a^ (days)	1 (9)	0 (7)	11.5 (23)	**< 0.001**
Postoperative ventral hernia	69	56 (13%)	13 (12%)	0.5
Entero-atmospheric fistula	26	17 (4%)	9 (8%)	0.06
Mortality	331	296 (45%)	35 (32%)	**0.011**

All variables with a *p* value < 0.05 were considered significant and are in bold

*TAC* temporary abdominal closure

^a^Values presented as median (IQR) or absolute numbers (%)

**Table 4:** Risk factors for delayed fascial closure and secondary healing

	Total *N* = 80	Delayed fascial closure *N* = 43	Secondary healing *N* = 37	*p* value	Odds ratio	95% CI
Indication for TAC						
DCS	36 (45.0%)	26 (60.5%)	10 (27%)	**0.003**	NS	
Abdominal sepsis	27 (33.8%)	9 (20.9%)	18 (48.6%)	**0.013**		
ACS	17 (21.3%)	8 (18.6%)	9 (24.3%)	NS		
Trauma	32 (40%)	22 (51%)	10 (27%)	**0.028**	NS	
Non-trauma	48 (60%)	21 (49%)	27 (73%)			
Surgeries before TAC	1 (2)	0 (1)	1 (2)	**0.001**	0.41	**0.24–0.72**
Surgeries during TAC	2 (3)	1 (1)	3 (4)	**0.002**	0.65	**0.49–0.87**
Total duration TAC(days)	7 (17)	3 (5)	18 (28)	**< 0.001**		

All variables with a *p* value < 0.05 were considered significant and are in bold

All data are expressed in median (IQR) or absolute numbers (%)

*NS* not statistically significant, *TAC* temporary abdominal closure

**Fig. 2:**
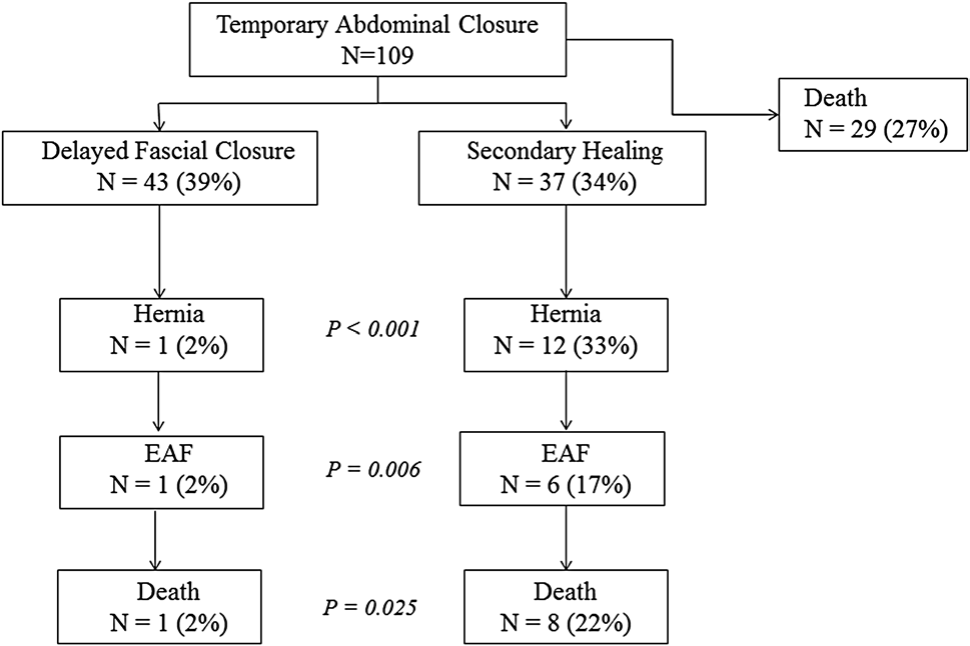
Outcome

When analyzing outcome in the TAC patient group, there was a significant difference in outcome between patients after delayed fascial closure and secondary healing. Patients who underwent secondary healing had higher mortality rates, developed more often entero-atmospheric fistulas and ventral hernias than patients in whom delayed fascial closure was successful (Fig. 2; Table 5).

**Table 5:** Outcome parameters following open abdomen treatment

	Total *N* = 80	Delayed fascial closure *N* = 43	Secondary healing *N* = 37	*p* value
Hospital LOS	46 (40)	38 (45)	56 (37)	**0.013**
ICU-LOS	10 (23)	9 (13)	16 (26)	**0.021**
Postoperative hernia	13 (16%)	1 (2.3%)	12 (33.3%)	**< 0.001**
Entero-atmospheric fistula	7 (8%)	1 (2.3%)	6 (16.7%)	**0.025**
Mortality	9 (11%)	1 (2.3%)	8 (21.6%)	**0.006**

All variables with a *p* value < 0.05 were considered significant and are in bold

All data are expressed in median (IQR) or absolute numbers (%)

*LOS* length of stay

The majority of patients who were treated with TAC in case of DCS could be managed with delayed fascial closure, in contrast with patients with abdominal sepsis, since most of them continued to secondary healing following TAC (Table 4). In abdominal compartment syndrome, both delayed fascial closure and secondary healing occurred in half of the patients (Table 4). Patients who underwent secondary healing needed more surgical interventions to finally close the abdomen compared to patients who underwent TAC for delayed fascial closure. Further, the total duration of TAC treatment was significantly longer in patients following secondary healing compared to patients who underwent successful delayed fascial closure (Table 4). When evaluating multivariate analysis (both forward and backward stepwise selection) for the ability of abdominal closure, only surgical interventions before TAC and during TAC were significant predictors. We excluded total duration of TAC for the multivariate analysis since this represents more or less an outcome variable and besides that it roughly corresponds to the interventions during TAC; therefore, we chose to only include one of these two variables in multivariate analysis. These results show that the odds for secondary healing were 2.5 times higher with every previous intervention and 1.5 times higher for every intervention during open abdominal treatment (Table 4).

## Discussion

Male gender and pH, as an indicator of the severity of physiological derangement of the patients, at the index operation were the only significant factors associated with possible failure of abdominal wall closure in our studied population, while patients with gastro-intestinal indication for surgery were less likely to end up with an open abdomen. In addition, the number of surgeries prior to TAC and the number of surgeries during TAC, but not the indication nor disease severity prior to the index laparotomy, were significant risk factors for the failure of delayed fascial closure.

The main reasons for open abdominal treatment were DCS in trauma (45%), followed by abdominal sepsis (34%) and abdominal compartment syndrome (21%). All abdominal sepsis patients had an open abdomen, because it could physically not be closed due to extensive swelling of the abdominal contents. None of them had their abdomen left open for a planned re-laparotomy. This is in accordance with Ruler et al. who have previously demonstrated that outcomes did not differ significantly following planned or on-demand re-laparotomy in patients with abdominal sepsis [7].

In our study, 27% of patients died before the abdomen could be closed. This high mortality rate, a reflection of the critically ill patients in whom the abdomen is frequently left open, is comparable to other studies [1, 5]. Further, an open abdomen itself is associated with multiple problems such as blood loss, fluid and electrolyte losses, respiratory problems and closure of the abdomen itself is challenging in most cases [1, 2, 4]. These critically ill patients are in a hypercatabolic state due to fluid and protein loss, causing nutritional insufficiency [1]. These patients must be adequately resuscitated to prevent hypovolemia with substitution of protein loss since this can lead to compromised wound healing, increase of infections and decreased survival [19–21]. Further, ventilatory problems occur frequently because an intact abdominal wall is required for adequate spontaneous ventilation [4]. Lastly, it is associated with local complications such as ventral hernia development due to fascial retraction and entero-atmospheric fistulas (EAF) [1–4].

Male gender and acidosis have shown to be associated with open abdominal treatment. This has not yet been illustrated in previous investigations. One could only speculate why male gender is prone to open abdominal treatment. Possibly, since male patients are more muscular than female patients, they might have a less compliant abdominal wall. Besides that, a large part of the female population was past the child-bearing age and might have had children which means that during pregnancy the abdominal wall has been already stretched, possibly making it more compliant in case of swelling of abdominal contents. Further, patients following gastro-intestinal surgery were less likely to undergo open abdomen treatment. This could possibly be explained by the fact that surgery is usually planned in case of gastro-intestinal disease, whereas patients who have a laparotomy following trauma had it in an acute setting.

It is also important to distinguish between patients who have an open abdomen as a consequence of physiological derangement, and patients who have an open abdomen because of local abdominal disease. In our study population, patients with abdominal sepsis were less likely to undergo delayed fascial closure compared to patients with an open abdomen after DCS, (mainly in trauma patients) and most of abdominal sepsis patients ended with a definitive open abdomen. This difference in abdominal closure determined by the origin of the open abdomen has been described before by Loftus et al. [5]. They investigated a group of 224 patients that underwent open abdominal treatment. In the patients who survived, the primary closure rate for trauma patients was much higher than in patients with abdominal sepsis (90% compared to 76%). Tolonen et al. [15] have investigated outcomes in patients following temporary abdominal closure for abdominal peritonitis only. In a group of 41 patients, they found a very high fascial closure rate of 92% [15]. In a systematic review of patients treated with temporary abdominal closure for abdominal sepsis, performed by Atema et al. [6], delayed fascial closure rates were more comparable to ours with a mean delayed fascial closure rate of 50.2%. Our study results showed that the number of abdominal surgical procedures prior to and during TAC was the only significant indicator for the possibility to eventually close the abdomen (by delayed fascial closure). This has been reported before by Atema et al. [6] who demonstrated in their systematic review that fewer re-explorations and shorter duration of temporary abdominal closure were associated with greater possibility of delayed fascial closure as well. This difference might be related to origin of TAC as well since patients following trauma are severely injured at the time of presentation and undergo immediate damage control surgery followed by open abdominal treatment. Generally, after a short period of resuscitation in ICU with only a few re-explorations, the abdomen can be closed by delayed fascial closure. This is in contrast with patients with abdominal sepsis, for example, who might have had previous abdominal surgeries and then undergo open abdominal treatment because of severely ill (contaminated) abdomen. These patients are more likely to have more re-explorations before the abdomen can finally be closed again, if it could be closed at all.

With the various complications that may occur following secondary healing, one should attempt to primarily close the abdomen. In our opinion, a structured approach, as listed in the flowchart, we use in our trauma department (Fig. 1), could contribute to favorable outcomes. We strive for a second look within 24–48 h and try immediately to close the fascia; if not feasible, we try again within the next 48 h. Other reports also mention this window of 48 h to close the abdomen, with increasing complication rates thereafter [2, 3]. When it becomes clear that delayed fascial closure is not feasible after two attempts, one should not continue TAC treatment, but change strategies to, for example, Ramirez technique [14], or vacuum-assisted wound closure combined with mesh-mediated fascial traction, a technique described by Petersson et al. [16]. They have introduced the combined use of mesh with the vacuum system, where the mesh is tightened with change of vacuum system every 2–3 days until the fascia could be closed again [16]. Rasilainen et al. [17] have evaluated this technique as well, with very high closure rates of 78% in 50 patients. In a recently published study by Salamone et al. [18], an even higher closure rate of 95% was found in a modified version of this technique.

Our study has some limitations inherent to the retrospective design of the study, conducted in a single institution. Due to this retrospective nature, our long-term data were dependent upon clinic visit data with follow-up varying from several days to months. Another limitation was the heterogeneity in treatment. There is no strict protocol in our hospital for open abdominal treatment after gastro-intestinal surgery and the decision to try to primarily close the abdomen or use a mesh is made at the surgeon’s discretion. Only in trauma patients there has been a strict protocolled approach for open abdominal treatment.

In conclusion, our results show that male sex and disease severity, as indicated by severe acidosis, were independent predictors of open abdominal treatment, while laparotomy in the context of gastro-intestinal pathology usually ends with a primary closed fascia. Furthermore, delayed fascial closure of the abdomen was possible in a small majority of the patients, with a significantly lower closure rate in patients following abdominal sepsis. However, the only independent predictor of delayed fascial closure was the quantity of abdominal surgical procedures before and during TAC treatment. Failure to achieve delayed fascial closure increases the risk of developing a ventral hernia and entero-atmospheric fistulas. We, therefore, advise to aim for delayed fascial closure as early as possible following a structured approach. In addition, we would advise to refrain from fruitless attempts at fascial closure, but rather try mesh-mediated vacuum closure or accept a definitive open abdomen. A potential ventral hernia could be repaired at a later stage.

## Abbreviations

DCS: Damage control surgery
ACS: Abdominal compartment syndrome
EAF: Entero-atmospheric fistula
TAC: Temporary abdominal closure
